# Regulation of Human Hepatic Drug Transporter Activity and Expression by Diesel Exhaust Particle Extract

**DOI:** 10.1371/journal.pone.0121232

**Published:** 2015-03-24

**Authors:** Marc Le Vee, Elodie Jouan, Bruno Stieger, Valérie Lecureur, Olivier Fardel

**Affiliations:** 1 Institut de Recherches en Santé, Environnement et Travail (IRSET), UMR INSERM U1085, Faculté de Pharmacie, 2 Avenue du Pr Léon Bernard, 35043 Rennes, France; 2 Department of Clinical Pharmacology and Toxicology, University Hospital, 8091 Zurich, Switzerland; 3 Pôle Biologie, Centre Hospitalier Universitaire, 2 rue Henri Le Guilloux, 35033 Rennes, France; University Paris Diderot-Paris 7, FRANCE

## Abstract

Diesel exhaust particles (DEPs) are common environmental air pollutants primarily affecting the lung. DEPs or chemicals adsorbed on DEPs also exert extra-pulmonary effects, including alteration of hepatic drug detoxifying enzyme expression. The present study was designed to determine whether organic DEP extract (DEPe) may target hepatic drug transporters that contribute in a major way to drug detoxification. Using primary human hepatocytes and transporter-overexpressing cells, DEPe was first shown to strongly inhibit activities of the sinusoidal solute carrier (SLC) uptake transporters organic anion-transporting polypeptides (OATP) 1B1, 1B3 and 2B1 and of the canalicular ATP-binding cassette (ABC) efflux pump multidrug resistance-associated protein 2, with IC50 values ranging from approximately 1 to 20 μg/mL and relevant to environmental exposure situations. By contrast, 25 μg/mL DEPe failed to alter activities of the SLC transporter organic cation transporter (OCT) 1 and of the ABC efflux pumps P-glycoprotein and bile salt export pump (BSEP), whereas it only moderately inhibited those of sodium taurocholate co-transporting polypeptide and of breast cancer resistance protein (BCRP). Treatment by 25 μg/mL DEPe was next demonstrated to induce expression of BCRP at both mRNA and protein level in cultured human hepatic cells, whereas it concomitantly repressed mRNA expression of various transporters, including OATP1B3, OATP2B1, OCT1 and BSEP. Such changes in transporter expression were found to be highly correlated to those caused by 2,3,7,8-tetrachlorodibenzo-p-dioxin (TCDD), a reference activator of the aryl hydrocarbon receptor (AhR) pathway. This suggests that DEPe, which is enriched in known ligands of AhR like polycyclic aromatic hydrocarbons, alters drug transporter expression via activation of the AhR cascade. Taken together, these data established human hepatic transporters as targets of organic chemicals containing in DEPs, which may contribute to their systemic effects through impairing hepatic transport of endogenous compound or drug substrates of these transporters.

## Introduction

Diesel exhaust particles (DEPs) are major and widely-distributed environmental air pollutants, originating from diesel engines [1]. They are usually composed of a center core of elemental carbon and adsorbed organic compounds, including polycyclic aromatic hydrocarbons (PAHs) and nitro-PAHs, and small amounts of sulfate, nitrate, metals, and other trace elements. They have sizes generally less than 1 μm and, as such, represent a mixture of fine (diameter below 2.5 μm), ultrafine (diameter below 100 nm) and nano particles (diameter below 50 nm) [2]. Human exposure to these DEPs is very frequent, especially in urban areas [3], and is thought to promote airway inflammation, asthma, cardiopulmonary diseases and lung cancer [4–6].

Even if toxic effects of DEPs primarily target the lung, thus reflecting that the major, if not exclusive, way of exposure to these pollutants is inhalation, systemic effects, including vascular and inflammatory effects, also occur [7–9]. This may be consistent with the passage across the pulmonary alveolar-capillary barrier of ultrafine DEPs [10] and/or of some organic or inorganic compounds primarily adsorbed on DEPs such as PAHs [11]. In this context, exposure to DEPs has been demonstrated to affect the liver, notably causing fatty changes, accumulation of lipid peroxidation products, activation of the leukotrienes-producing 5-lipoxygenase pathway and up-regulation of inflammatory cytokines [12, 13]. The drug metabolizing enzymes cytochrome P-450 (CYP) 1A1 and CYP1B1 and the antioxidant enzyme NAD(P)H-quinone oxidoreductase 1 are also induced in hepatic cells exposed to DEP extract (DEPe) and in the liver of rodents exposed to DEPs [14–16]. Such data indicate that these pollutants, like other inhaled deleterious contaminants such as cigarette smoke [17, 18], may alter hepatic detoxification pathways, probably through activation of the aryl hydrocarbon receptor (AhR) pathway [19].

It is noteworthy that hepatic drug detoxifying pathways implicate not only enzymes like CYPs, but also membrane drug transporters [20]. These transporters, that belong to the solute carrier (SLC) or the ATP-binding cassette (ABC) transporter families, mediate uptake of drugs at the sinusoidal pole of hepatocytes and their efflux into the bile at the canalicular pole [21]. Some of them, especially the ABC transporter P-glycoprotein (ABCB1) and the breast cancer resistance protein (BCRP/ABCG2), have been shown to be regulated by inhalable chemical contaminants, including cigarette smoke extract [22] and PAHs [23–25]. In the same way, DEPs have been shown to induce expression of P-glycoprotein, BCRP and multidrug resistance-associated protein (MRP) 2 (ABCC2) at the blood-brain barrier [26]. By contrast, whether DEP-adsorbed chemicals may affect activity and/or expression of hepatic drug transporters remains unknown. The present study was therefore designed to get insights about this point. Our data demonstrate that organic DEPe markedly inhibited activity of organic anion-transporting polypeptides (OATPs/SLCOs) and of MRP2 and induced BCRP expression in cultured human hepatocytes and hepatocyte-like cells. Such changes may contribute to systemic effects of DEPs through impairing hepatic transport of endogenous compounds or drugs substrates of these transporters.

## Materials and Methods

### Chemicals

DEPe used in the study was the Standard Reference Material 1975 (SRM 1975), purchased by the National Institute of Standards and Technology (NIST) (Gaithersburg, MD, USA). It corresponds to a dichloromethane extract of filter-collected combustion particulate matter from operating forklifts with diesel engines [27]; some of its chemical components have been characterized in the certificate of analysis provided by NIST [28]. Dichloromethane was evaporated under nitrogen and the final residue was dissolved in dimethyl sulfoxide (DMSO) for cell exposure. Final concentration of DMSO did not exceed 0.2% (vol/vol); control cultures received the same dose of solvent as for treated counterparts. Verapamil, probenecid, fumitremorgin C, bromosulfophtalein, fluorescein, fluoranthene, phenanthrene, benzo[b]fluoranthene, chrysene, 1-nitropyrene and 1,2-naphtoquinone were provided by Sigma-Aldrich (Saint-Quentin Fallavier, France), whereas carboxy-2,7-dichlorofluorescein (CF) diacetate and Hoechst 33342 were from Life Technologies (Saint Aubin, France) and 2,3,7,8-tetrachlorodibenzo-*p*-dioxin (TCDD) from Cambridge Isotope Laboratories (Cambridge, MA). [^3^H(G)] taurocholic acid (sp. act. 1.19 Ci/mmol), [6,7-^3^H(N)] estrone-3-sulfate (E3S) (sp. act. 57.3 Ci/mmol) and [1–^14^C] tetra-ethylammonium (TEA) (sp. act. 2.4 mCi/mmol) were from Perkin-Elmer (Boston, MA). All other chemicals were commercial products of the highest purity available.

### Cell culture

Human hepatocyte suspensions were provided by the Biological Resource Center (University Hospital, Rennes, France), which has obtained the authorization N°DC-2008-630 from the French Ministry of Health to collect hepatic resections from the digestive surgery department and then to isolate and deliver the hepatocytes used in this study. All of liver fragment donors provided a written informed consent to participate in the study. Basic information related to liver fragment donors, including age, sex and type of disease, was collected by the Biological Resource Center and is available. The authors did not interact with the donors of hepatocytes and have no direct access to their clinical and biological data. Hepatocytes, initially prepared by enzymatic dissociation of histologically-normal liver fragments [29], were seeded on plastic dishes at a density of 2 × 10^5^ cells/cm^2^ in Williams' E medium (Invitrogen, Cergy-Pontoise, France), supplemented with 10% (vol/vol) fetal calf serum (Perbio Sciences, Brébieres, France), 5 μg/mL bovine insulin (Sigma-Aldrich), 100 IU/mL penicillin, 100 μg/mL streptomycin, and 2 mM glutamine (Invitrogen). After 24 h, this seeding medium was discarded, and primary hepatocytes were routinely cultured in the fetal calf serum-containing Williams' E medium defined above and supplemented with 5 × 10^−5^ M hydrocortisone hemisuccinate (Upjohn, Paris La Défense, France) and 2% (vol/vol) DMSO, as reported previously [30]. All experimental procedures complied with French laws and regulations and were approved by the National Ethics Committee.

Human highly-differentiated hepatoma HepaRG cells, which are well-recognized as surrogates for human hepatocytes in drug detoxification pathway studies [31], were routinely cultured in Williams' E medium (Life Technologies) supplemented with 10% (vol/vol) fetal calf serum, 100 IU/mL penicillin, 100 μg/mL streptomycin, 5 μg/mL insulin, 2 mM glutamine, and 5 x 10^−5^ M hydrocortisone hemisuccinate. Additional culture for two weeks in the same medium supplemented with 2% (vol/vol) DMSO was performed in order to get a full hepatocytic differentiation of the cells [32].

P-glycoprotein-overexpressing mammary MCF-7/R cells [33] and MRP2-expressing human hepatoma HuH-7 cells [34] were cultured in Dulbecco’s modified Eagle medium (DMEM) (Life Technologies), supplemented with 10% (vol/vol) fetal calf serum, 100 IU/mL penicillin and 100 μg/mL streptomycin. BCRP-transfected HEK 293 cells [35], kindly donated by Dr X. Decleves (Faculty of Pharmacy, University Paris-Descartes, Paris, France), were cultured in DMEM supplemented with 10% (vol/vol) fetal calf serum, 100 IU/mL amoxicillin, 100 μg/mL erythromycin and 200 μg/mL G418. OATP1B1 (SLCO1B1)-, OATP1B3 (SLCO1B3)- and OATP2B1 (SLCO2B1)-transfected CHO cells were cultured in DMEM containing 100 IU/mL penicillin, 100 μg/mL streptomycin, 10% (vol/vol) fetal calf serum and 500 μg/mL G418, as previously reported [36].

### Transporter activity assays

#### Uptake SLC transporter activity

The effects of DEPe on sodium taurocholate co-transporting polypeptide (NTCP/SLC10A1), OATP and organic cation transporter (OCT) 1 (SLC22A1) activities were analysed through determining intracellular accumulation of reference substrates of these sinusoidal influx transporters using a well-defined transport medium [37]. Briefly, for NTCP activity, cells were incubated at 37°C with 43.4 nM [^3^H] taurocholate for 10 min, in the absence or presence of sodium or DEPe; for OATP activity, cells were incubated at 37°C for 6 min with 3.4 nM [^3^H] E3S or 10 μM fluorescein, in the absence or presence of DEPe or reference OATP inhibitors (probenecid or bromosulfophtalein) [38, 39]; for OCT1 activity, HepaRG cells were incubated at 37°C with 40 μM [^14^C] TEA for 5 min, in the absence or presence of DEPe or a reference OCT1 inhibitor (verapamil) [40]. After washing in phosphate-buffered saline (PBS), cells were lysed and accumulation of substrates was determined through scintillation counting (for taurocholate, E3S and TEA) or through spectrofluorimetry (for fluorescein) using a SpectraMax Gemini SX spectrofluorometer (Molecular Devices, Sunnyvale, CA) (Excitation and emission wavelengths were 485 and 535 nm, respectively). Data were expressed as amount of intracellular substrate/mg protein. For OATPs, data were also expressed as % of E3S uptake found in control cells or as % of OATP activity, according to the following equation:

$$\%\;\text{OATP activity}=\frac{\left({\text{Accumulation}}_{\text{DEPe}}-{\text{Accumulation}}_{\text{Reference inhibitor}}\right)\times 100}{{\text{Accumulation}}_{\text{Control}}\text{−}{\text{Accumulation}}_{\text{Reference inhibitor}}}$$

with Accumulation_DEPe_ = substrate accumulation in the presence of DEPe, Accumulation_Control_ = substrate accumulation in control cells, Accumulation_Reference inhibitor_ = substrate accumulation in the presence of a reference OATP inhibitor.

#### Efflux ABC transporter activity

The effects of DEPe on P-glycoprotein activity were analysed in P-glycoprotein-expressing MCF-7/R cells, through measuring intracellular accumulation of the P-glycoprotein substrate rhodamine 123 [41]. Briefly, MCF-7/R cells were incubated at 37°C with 5.25 μM rhodamine 123 for 30 min, in the presence or absence of DEPe or a reference P-glycoprotein inhibitor (verapamil) [42]. After washing in PBS, cells were lysed and intracellular accumulation of rhodamine 123 was determined by spectrofluorimetry (Excitation and emission wavelengths were 485 and 535 nm, respectively). Data were expressed as % of rhodamine 123 accumulation in control cells.

The effects of DEPe on MRP2 activity were analysed in MRP2-expressing HuH-7 cells, through measuring intracellular accumulation of the MRP2 substrate CF [43]. Briefly, HuH-7 cells were incubated at 37°C with 3 μM CF diacetate for 30 min, in the presence or absence of DEPe or a reference MRP2 inhibitor (probenecid) [43]. Intracellular accumulation of CF was determined by spectrofluorimetry as reported above. Data were expressed as % of CF accumulation in control cells or as % of MRP2 activity according to the following equation:

$$\%\;\text{MRP2 activity}=\frac{\left({\text{Accumulation}}_{\text{Reference inhitor}}-{\text{Accumulation}}_{\text{DEPe}}\right)\times 100}{{\text{Accumulation}}_{\text{Reference inhibitor}}-{\text{Accumulation}}_{\text{Control}}}$$

with Accumulation_DEPe_ = CF accumulation in the presence of DEPe, Accumulation_Control_ = CF accumulation in control cells, Accumulation_Reference inhibitor_ = CF accumulation in the presence of a reference MRP2 inhibitor.

The effects of DEPe on BCRP activity were determined in BCRP-HEK 293 cells through measuring intracellular retention of the BCRP substrate Hoechst 33342 [44]. Briefly, BCRP-HEK 293 cells were first loaded at 37°C with 16.2 μM Hoechst 33342 for 30 min. After washing in PBS, cells were re-incubated in Hoechst 33342-free medium at 37°C for 90 min in the absence or presence of DEPe or 10 μM fumitremorgin C, used here as a reference inhibitor for BCRP [45]. Intracellular retention of Hoechst 33342 was determined by spectrofluorimetry as described above (Excitation and emission wavelengths were 355 and 460 nm, respectively). Data were expressed as % of initial Hoechst 33342 loading.

The effects of DEPe on bile salt export pump (BSEP/ABCB11) activity were analysed in primary human hepatocytes, through measuring BSEP-mediated secretion of taurocholate into bile canaliculi-like structures as previously reported [46]. Briefly, hepatocytes were first incubated for 10 min at 37°C with transport assay buffer containing Ca^2+^ or with the same buffer, except that 1.8 mM CaCl_2_ was withdrawn and 100 μM EGTA was added; incubation with this Ca^2+^-free buffer promotes disruption of tight junctions and opening of bile canaliculi networks [46]. Buffers were then removed and hepatocytes were further incubated for 10 min at 37°C in transport assay medium containing 43.4 nM [^3^H] taurocholate in the absence or presence of DEPe. After washing with PBS, accumulations of taurocholate into cells + bile canaliculi (Ca^2+^-containing conditions) and into cells only (Ca^2+^-free conditions) were determined by scintillation counting. Biliary excretion index (BEI) reflecting canalicular secretion of taurocholate was calculated using the following equation [47]:

$$\text{BEI}=\frac{\text{Accumulation}\;\left(\text{Cells}+\text{Bile canaliculi}\right)-\text{Accumulation}\;\left(\text{Cells}\right)}{\text{Accumulation}\;\left(\text{Cells}+\text{Bile canaliculi}\right)}\times 100$$

### Protein concentration determination

Protein concentration in cell samples was determined using the Bradford assay as previously described [48].

### RNA isolation and analysis

Total RNAs were extracted using the TRI reagent (Sigma-Aldrich). RNA was then subjected to reverse transcription-quantitative polymerase chain reaction (RT-qPCR), using the RT kit from Applied Biosystems (Foster City, CA), the fluorescent dye SYBR Green methodology and an ABI Prism 7300 detector (Applied Biosystems), as previously described [49]. Gene-specific primers for drug transporters, CYP1A1, CYP1B1, aldehyde deshydrogenase 3A1 (ALDH3A1) and 18S RNA were used exactly as previously reported [49–51]. The specificity of each gene amplification was verified at the end of qPCR reactions through analysis of dissociation curves of the PCR products. Amplification curves were analysed with ABI Prism 7000 SDS software, using the comparative cycle threshold method. Relative quantification of the steady-state target mRNA levels was calculated after normalization of the total amount of cDNA tested to the 18S RNA endogenous reference using the 2^(−ΔCt)^ method. Data were finally expressed as % of values found in untreated control cells or in arbitrary units relative to 18S RNA content as previously described [52].

### Western-blot analysis

Crude membrane extracts were prepared as previously reported [53]. Proteins were then separated on SDS polyacrylamide gels and electrophoretically transferred to nitrocellulose membranes. After blocking in Tris-buffered saline containing 4% bovine serum albumin, membranes were incubated overnight at 4°C with primary antibodies directed against OATP1B1, OATP2B1 [54], P-glycoprotein, BCRP (Alexis Biochemicals, Lausen, Switzerland), MRP2 or MRP3 (Millipore Bioscience Research Reagents, Temecula, CA). Peroxidase-conjugated monoclonal antibodies were thereafter used as secondary antibodies. After washing, immuno-labelled proteins were visualized by chemiluminescence. Gel loading and transfer was checked up by staining membranes with Ponceau red [55]. Intensities of antibody-stained bands and Ponceau red-stained lanes were measured by densitometry using ImageJ 1.40g software (National Institute of Health, Besthesda, MA), allowing to normalise antibody-related staining to Ponceau red-labeling.

### Calculation of PAH concentrations in DEPe solutions

PAH concentrations in DEP solutions were calculated from mass fractions for selected PAHs reported in SRM 1975, according to the following equation:

$$\left[\text{PAH}\right]=\frac{\left({\text{Mass fraction}}_{\text{PAH}}\times \left[\text{DEPe}\right]\right)\times 1000}{{\text{Molecular weight}}_{\text{PAH}}}$$

### Statistical analysis

Quantitative data were usually expressed as means ± SEM. They were statistically analysed using ANOVA followed by a Dunnett’s post-hoc test, Student’s t-test or nonparametric Spearman’s rank correlation method. The criterion of significance was *p* < 0.05. Half maximal inhibitory concentration (IC_50_) values were determined using GraphPad Prism software (GraphPad Software, La Jolla, CA), through nonlinear regression based on the four parameter logistic function.

## Results

### Effects of DEPe on hepatic SLC transporter activity

**Fig 1:**
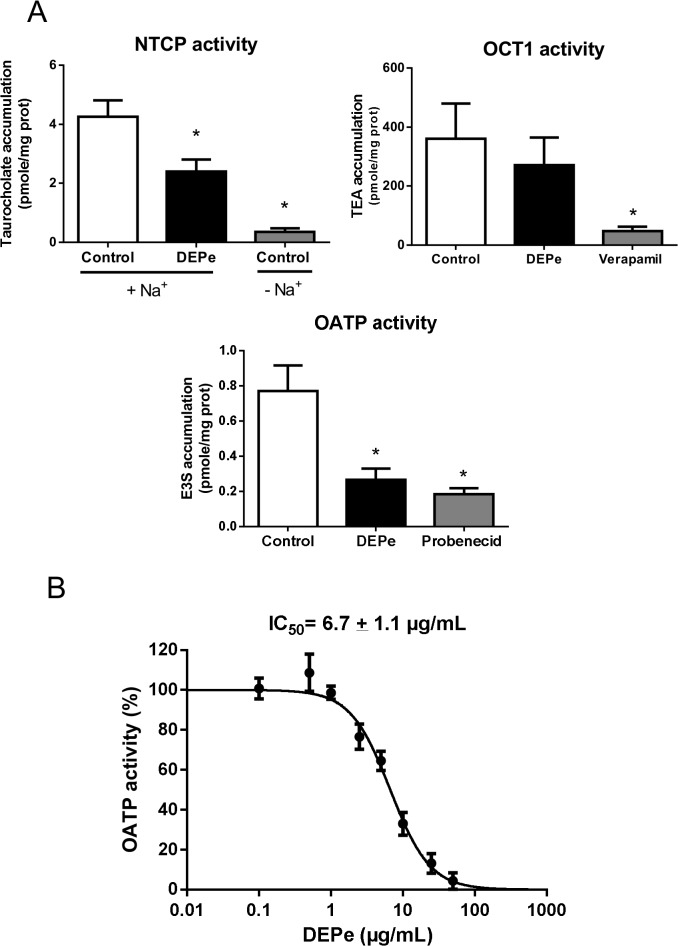
Effects of DEPe on NTCP, OATP and OCT1 transport activity in primary human hepatocytes. (A) NTCP-mediated uptake of taurocholate, OATPs-mediated uptake of E3S and OCT1-mediated uptake of TEA in primary human hepatocytes were determined in the absence or presence of 25 μg/mL DEPe as reported in the Material and Methods. Reference inhibition of NTCP, OATP and OCT1 activity was obtained in parallel through withdrawal of sodium (NTCP inhibition) or addition of 2 mM probenecid (OATP inhibition) or of 50 μM verapamil (OCT1 inhibition). Data are expressed as substrate accumulation/mg protein and are the means ± SEM of at least three independent experiments. *, p<0.05 when compared to control cells (ANOVA followed by a Dunnett’s post-hoc test). (B) OATPs-mediated transport of E3S was measured in the absence or presence of various concentrations of DEPe (from 0.1 to 50 μg/mL) or of the reference OATP inhibitor probenecid. Data are expressed as % of OATP activity calculated as described in Materials and Methods and are the means ± SEM of five independent assays. DEPe IC_50_ value is indicated at the top of the graph.

The potential direct effects of DEPe on uptake SLC drug transporter activities were first analysed in primary human hepatocytes. For this purpose, DEPe was added at a 25 μg/mL concentration, that likely corresponds to a 4.92 μg/cm^2^ equivalent DEP dose according to previous conversions of *in vitro* DEPe/DEP dose to DEP dose/unit surface area [56] and that is in the range of DEPe/DEP concentrations commonly retained for *in vitro* studies [57–59]. As shown in Fig. 1A, addition of DEPe was found to significantly, but rather moderately, decrease uptake of the NTCP substrate taurocholate in primary human hepatocytes; by contrast, withdrawal of sodium nearly fully blocked taurocholate accumulation, in agreement with the sodium-dependence of taurocholate transport by NTCP [60]. In contrast to the reference OCT1 inhibitor verapamil [40], DEPe failed to alter uptake of the OCT1 substrate TEA in primary human hepatocytes (Fig. 1A). DEPe, as well as the reference OATP inhibitor probenecid [38], was next shown to strongly inhibit uptake of the OATP substrate E3S. This inhibition of OATP activity in primary human hepatocytes by DEPe was concentration-dependent, with a DEPe IC_50_ value of 6.7 ± 1.1 μg/mL (Fig. 1B).

**Fig 2:**
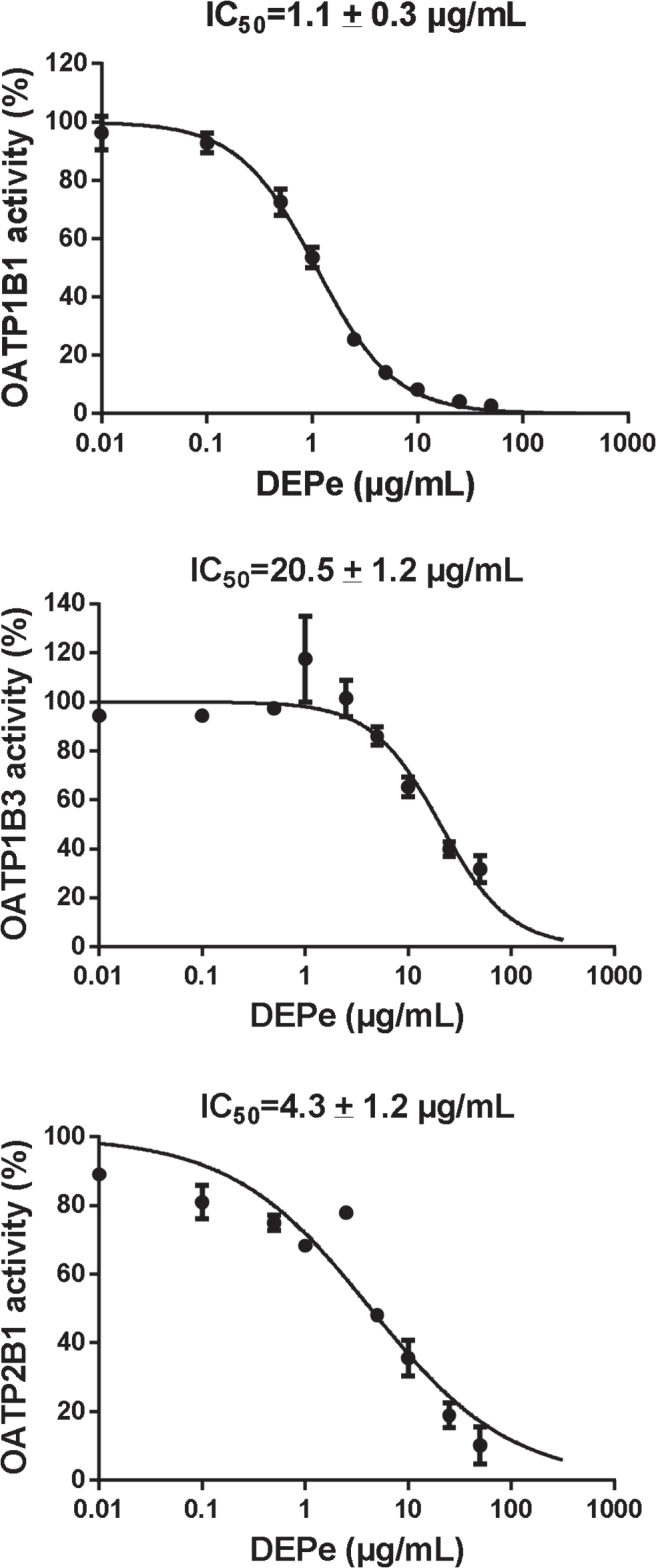
Concentration-dependent inhibition of OATP1B1, OATP1B3 and OATP2B1 activity by DEPe. OATP1B1- and OATP2B1-mediated transport of E3S and OATP1B3-mediated transport of fluorescein were determined in OATP-transfected CHO cells in the absence or presence of various concentrations of DEPs (from 0.1 to 50 μg/mL) or of the reference inhibitors 100 μM bromosulfophtalein (for OATP1B1 and OATP2B1) or 10 mM probenecid (for OATP1B3). Data are expressed as % of OATP activity calculated as described in Materials and Methods and are the means ± SEM of three independent assays. DEPe IC_50_ values are indicated at the top of the graphs.

Because human hepatocytes express several OATP transporters at their sinusoidal pole, *i*.*e*., OATP1B1, OATP1B3 and OATP2B1 [21], we investigated whether these different OATPs were directly inhibited by DEPe using OATP-transfected CHO cells and E3S as a reference substrate for OATP1B1 and OAPTP2B1 [61] and fluorescein as a reference substrate for OATP1B3 [62]. As shown in Fig. 2, DEPe blocked transport of E3S by both OATP1B1 and OATP2B1 in a concentration-dependent manner, OATP1B1 being more sensitive to the inhibitory effects of DEPe than OATP2B1 (IC_50_ were 1.1 ± 0.3 μg/mL and 4.3 ± 1.2 μg/mL, for OATP1B1 and OATP2B1, respectively). DEPe also inhibited OATP1B3-mediated transport of fluorescein, with a higher IC_50_ (20.5 ± 1.2 μg/mL) than those reported above for the inhibition of OATP1B1- and OATP2B1-mediated transport of E3S (Fig. 2).

### Effects of DEPe on ABC transporter activity

**Fig 3:**
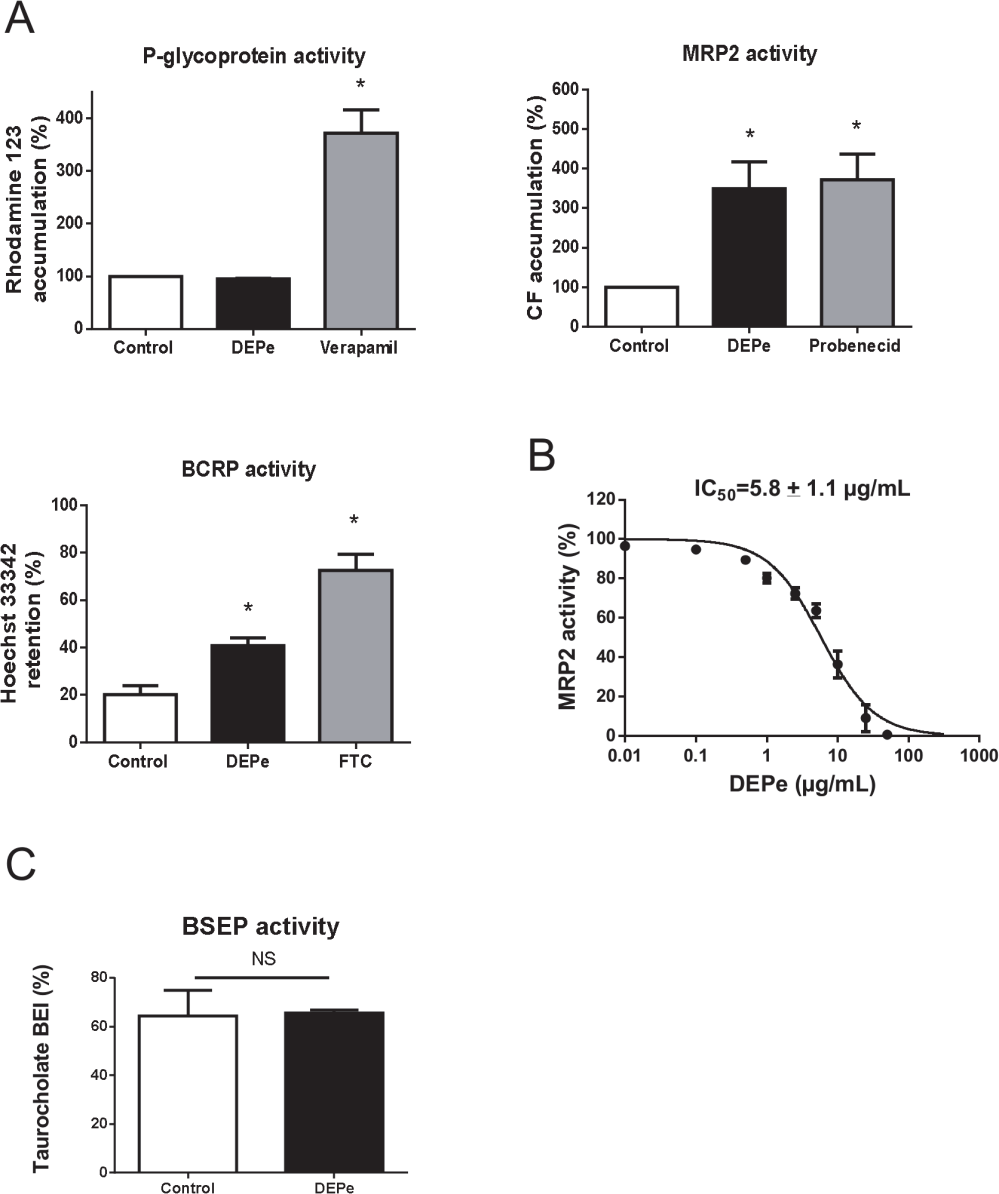
Effects of DEPe on P-glycoprotein, BCRP, MRP2 and BSEP transport activity. (A) P-glycoprotein, BCRP and MRP2 activities were determined through measurement of rhodamine 123 accumulation, Hoechst 33342 retention and CF accumulation in transporter-expressing cell lines in the absence or presence of 25 μg/mL DEPe as reported in the Material and Methods. Reference inhibition of transporter activity was obtained in parallel through addition of 50 μM verapamil (P-glycoprotein inhibition), 10 μM fumitremorgin C (FTC) (BCRP inhibition) or 10 mM probenecid (OATP inhibition). Data are expressed as % of dye accumulation (P-glycoprotein and MRP2 activities) or dye retention (BCRP activity) in control untreated cells; they are the means ± SEM of at least three independent experiments. *, p<0.05 when compared to control cells (ANOVA followed by a Dunnett’s post-hoc test). (B) CF accumulation was measured in HuH-7 cells in the absence or presence of various concentrations of DEPe (from 0.1 to 50 μg/mL) or of the reference OATP inhibitor probenecid. Data are expressed as % of MRP2 activity calculated as described in Materials and Methods and are the means ± SEM of four independent assays. DEPe IC_50_ value is indicated at the top of the graph. (C) BEI for taurocholate, reflecting BSEP activity, was determined in primary human hepatocytes in the absence or presence of 25 μg/mL DEPe, as reported in Materials and Methods. Data are the means ± SEM of three independent experiments. NS, not significant (Student’s t-test).

The potential direct effects of DEPe on ABC drug transporter activities were analysed in ABC-transporter overexpressing cells (for P-glycoprotein, MRP2 and BCRP) and in primary human hepatocytes (for BSEP). As shown in Fig. 3A, 25 μg/mL DEPe failed to induce accumulation of the P-glycoprotein substrate rhodamine 123 in P-glycoprotein-overexpressing MCF7/R cells, in contrast to the reference P-glycoprotein inhibitor verapamil [42]. Such data most likely indicate that DEPe did not inhibit P-glycoprotein-mediated efflux of rhodamine 123. DEPe was found to enhance retention of the BCRP substrate Hoechst 33342 in BCRP-transfected HEK 293 cells. This increase was however lower than that triggered by the reference BCRP inhibitor fumitremorgin C and DEPe failed to fully prevent loss of the dye during the efflux period (Fig. 3A), thus suggesting that DEPe altered BCRP-mediated efflux of the dye in a rather moderate manner. By contrast, DEPe (25 μg/mL) markedly induced accumulation of CF in MRP2-expressing HuH-7 cells by a 3.5-fold factor (Fig. 3A); the reference MRP2 inhibitor probenecid also similarly increased CF accumulation. Such data indicated that DEPe was able to strongly block MRP2-mediated efflux of CF. This inhibitory effect of DEPe towards MRP2 activity was moreover demonstrated to be concentration-dependent, with an IC_50_ value of 5.8 ± 1.1 μg/mL (Fig. 3B). The effects of DEPe on BSEP-mediated canalicular secretion of taurocholate was characterized through determining BEI for taurocholate in primary human hepatocytes (Fig. 3C). Similar BEI values, around 60%, were found in control and DEPe-exposed hepatocytes, thus likely indicating that DEPe failed to impair BSEP activity.

### Effects of DEPe-containing chemicals on OATP1B1 and MRP2 activity

**Fig 4:**
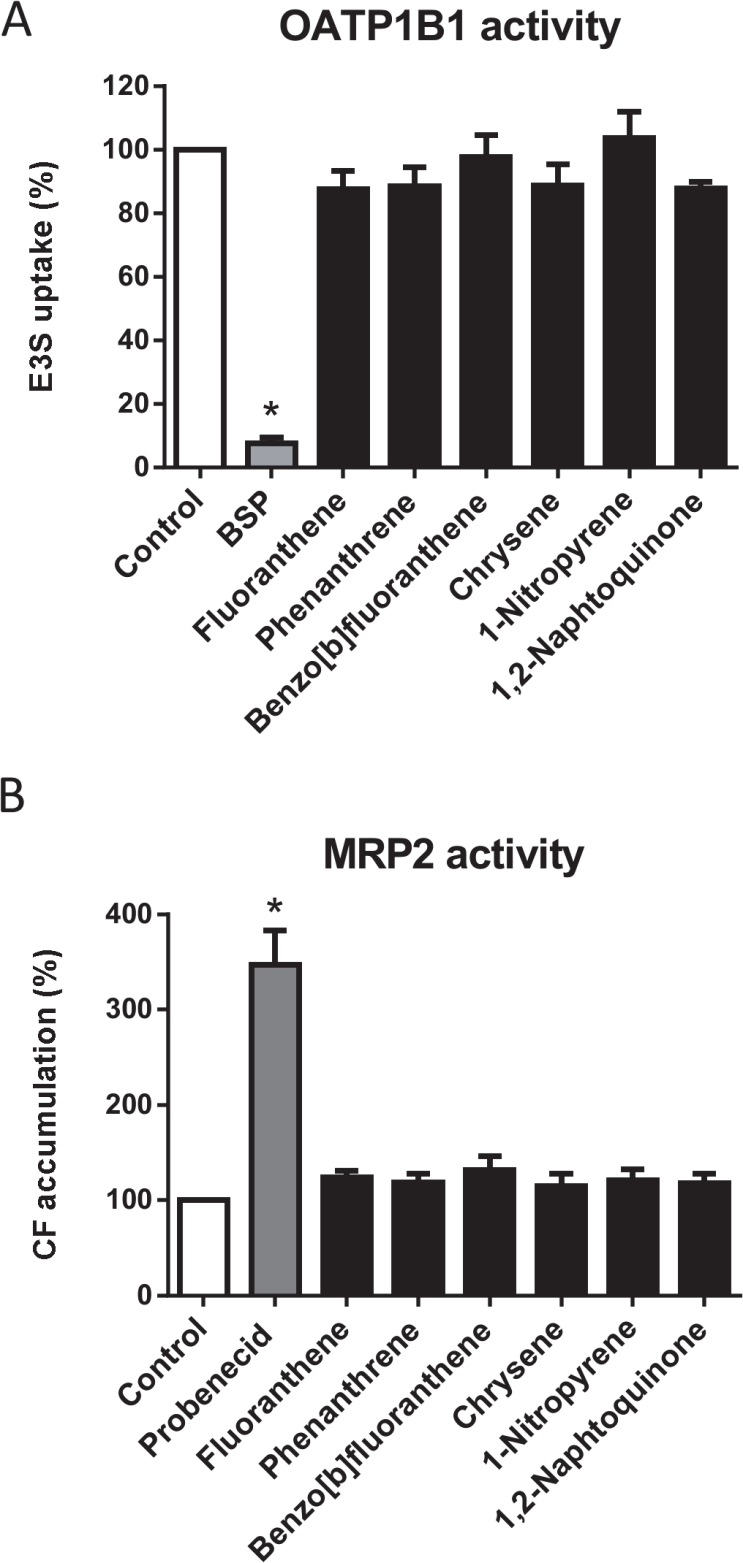
Effects of various DEPe-contained PAHs on OATP1B1 and MRP2 activity. (A) OATP1B1-mediated transport of E3S was determined in OATP1B1-transfected CHO cells in the absence or presence of the reference OATP inhibitor bromosulfophtalein (BSP) (100 μM) or of various DEPe-contained PAHs or PAH derivatives, each being used at 10 μM. Data are expressed as % of E3S accumulation in control untreated cells and are the means ± SEM of three independent assays. *, p<0.05 when compared to control cells (ANOVA followed by a Dunnett’s post-hoc test). (B) Accumulation of the MRP2 substrate CF was measured in HuH-7 cells in the absence or presence of DEPe (25 μg/mL) or of various DEPe-contained PAHs or PAH derivatives, each being used at 10 μM. Data are expressed as % of CF accumulation in control untreated cells and are the means ± SEM of three independent assays. *, p<0.05 when compared to control cells (ANOVA followed by a Dunnett’s post-hoc test).

Previous chemical characterization of DEPe used in the present study has revealed the presence of various PAHs and nitro-PAHs as main components of DEPe, as indicated by the certificate of analysis provided by NIST [28]. Because some PAHs, including PAH metabolites, have been shown to interfere with drug transporters, especially MRP2 [63], we wondered whether these compounds may be involved in DEPe-mediated inhibition of OATP1B1 and MRP2 activity. For such a purpose, we focused on some PAHs among the most concentrated ones present in DEPe, such as phenanthrene, fluoranthene, benzo[b]fluoranthene, chrysene and 1-nitropyrene [28]. The concentrations of these PAHs in DEPe solutions inhibiting 50% of OATP1B1 (Fig. 2) or MRP2 (Fig. 3B) activity were calculated from concentrations reported in native DEPe [28] and are indicated in Table 1. None of these PAHs, used at a 10 μM concentration much higher than those calculated in IC_50_-based DEPe solutions (that are in the 0.01–0.5 nM range, as shown in Table 1), was found to inhibit E3S accumulation in OATP1B1-transfected HEK 293 cells (Fig. 4A) or to enhance CF accumulation in MRP2-expressing HuH-7 cells (Fig. 4B), indicating a lack of inhibition of OATP1B1 or MRP2. The PAH derivative 1,2-naphtoquinone, whose presence has been reported in DEPs at a significant level [64], also failed to alter OATP1B1- and MRP2-related transport (Fig. 4A and Fig. 4B).

**Table 1:** Concentrations of selected PAHs in native DEPe and DEPe solutions.

PAH	Molecular weight	PAH concentration		
		Native DEPe^a^	DEPe solution	
			1.1 μg/mL (IC_50_/OATP1B1)	5.8 μg/mL (IC_50_/MRP2)
Phenanthrene	178.2 g	8.00 mg/kg	0.049 nM	0.26 nM
Fluoranthene	202.26 g	13.5 mg/kg	0.073 nM	0.39 nM
Benzo[b]fluoranthene	252.31 g	3.20 mg/kg	0.014 nM	0.073 nM
Chrysene	228.9 g	1.95 mg/kg	0.009 nM	0.050 nM
1-Nitropyrene	247.25 g	16.59 mg/kg	0.074 nM	0.39 nM

^a^Data for native DEPe are fraction mass reported in the certificate of analysis for SRM 1975 provided by NIST [28]

### Effects of DEPe on drug transporter expression

**Fig 5:**
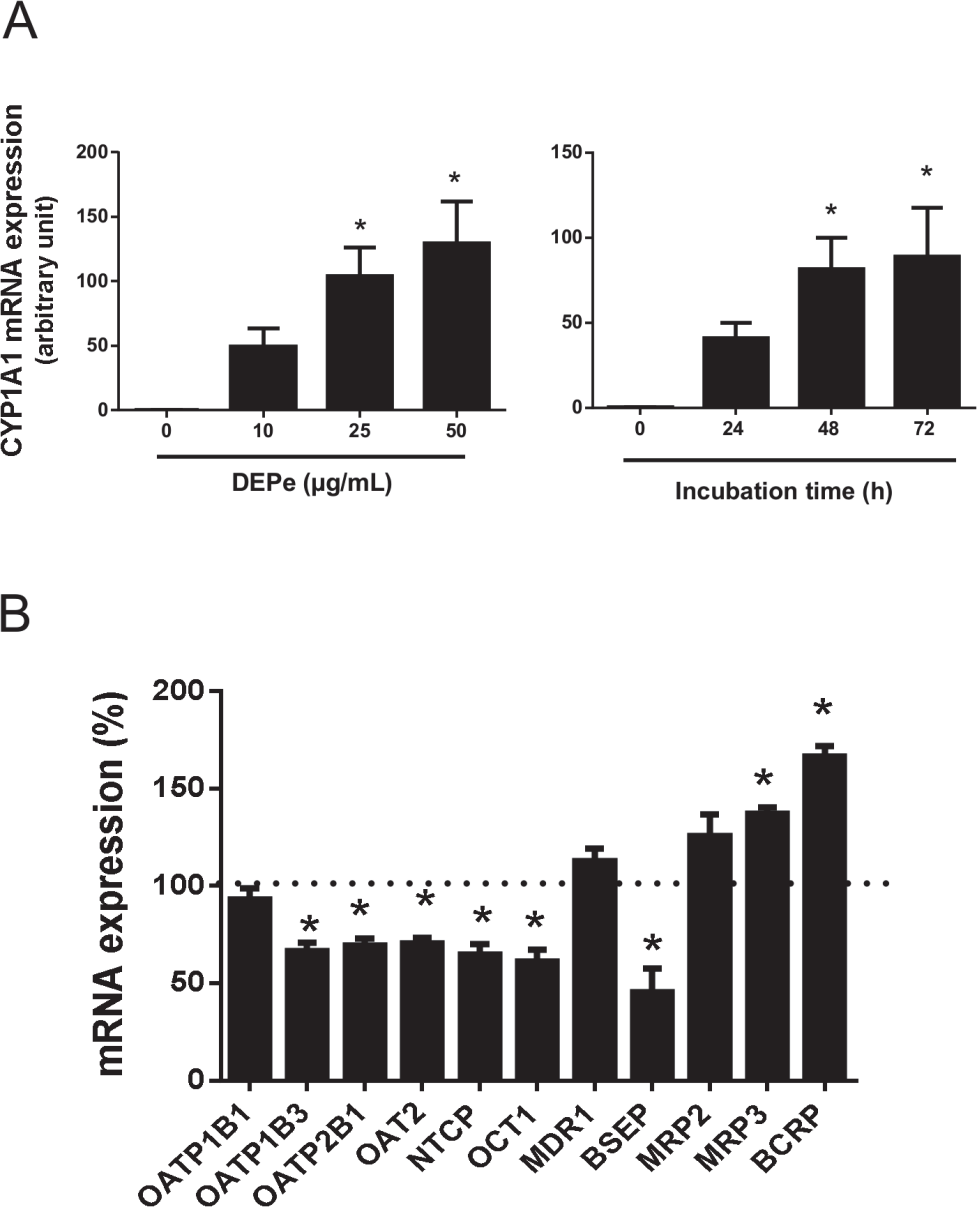
Regulation of drug transporter mRNA expression by DEPe. (A) Human highly-differentiated HepaRG cells were either untreated or exposed to various concentrations of DEPe for 48 h (right panel) or to 25 μg/mL DEPe for various exposure time (left panel). CYP1A1 mRNA expression was analysed by RT-qPCR. Data are expressed in arbitrary units relative to 18S RNA content and are the means ± SEM of three independent assays. *, p<0.05 when compared to control cells (ANOVA followed by a Dunnett’s post-hoc test). (B) HepaRG cells were either untreated or exposed to 25 μg/mL DEPe for 48 h. Drug transporter mRNA expression was evaluated by RT-qPCR. Data are expressed as % of transporter expression found in control untreated cells, arbitrary set at 100% and indicated by a dotted line; they are the means ± SEM of three independent assays. *, p<0.05 when compared to control cells (ANOVA followed by a Dunnett’s post-hoc test).

Human hepatoma HepaRG cells, that have been demonstrated to represent a convenient alternative to the use of primary human hepatocytes for transporter regulation studies [31, 65], were first exposed to various concentrations of DEPe (from 10 μg/mL to 50 μg/mL) for 48 h. Such treatments did not trigger any obvious toxicity in HepaRG cells as shown by light microscopic analysis and iodure propidium/Hoechst 33342 labeling of necrotic/apoptotic cells (data not shown). DEPe exposure increased mRNA expression of CYP1A1, a known reference target for DEPs (58). The induction was dose-dependent, *i*.*e*., it was higher for the DEPe concentrations of 25 μg/mL and 50 μg/mL than that for 10 μg/mL DEPe (Fig. 5A). DEPe used at 25 μg/mL was shown to similarly induce CYP1A1 expression for a 48 h-72 h treatment time; a shorter exposure time (24 h) was less active (Fig. 5A). The dose of 25 μg/mL DEPe and a treatment time of 48 h were consequently retained for analyzing the effects of DEPe on hepatic drug transporter expression. As shown in Fig. 5B, such an exposure to DEPe of HepaRG cells resulted in a down-regulation of mRNA expression of various SLC drug transporters including OATP1B3, OATP2B1, organic anion transporter 2 (OAT2/SCL22A7), OCT1 and NTCP. Repression fold-factors, *i*.*e*., the ratio expression in untreated control cells versus expression in DEPe-exposed cells, ranged from 1.42 (for OAT2) to 1.63 (for OCT1) and were rather moderate. The ABC efflux pump BSEP was also repressed by DEPe exposure, by a 2.21-fold factor (Fig. 5B). By contrast, MRP3 and BCRP mRNA levels were significantly induced by DEPe treatment, by 1.37- and 1.66-fold factors, respectively, whereas OATP1B1, MDR1 and MRP2 mRNA expression remained unchanged (Fig. 5B).

**Fig 6:**
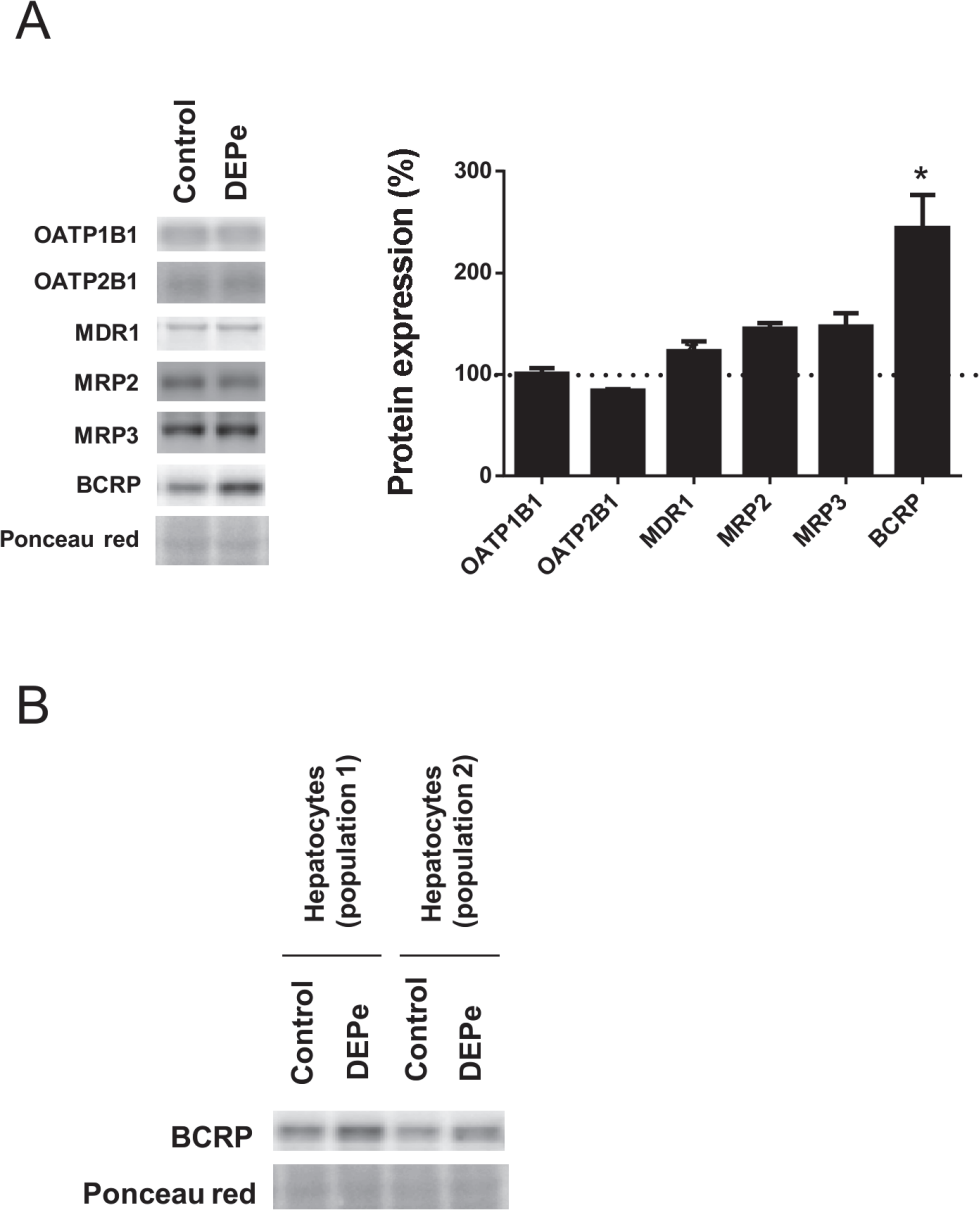
Regulation of drug transporter protein expression by DEPe. (A) HepaRG cells were either untreated (control) or exposed to 25 μg/mL DEPe for 48 h. Transporter protein content was then determined by Western-blot analysis. Left panel, a representative blot is shown for each transporter. Right panel, for each transporter, data were quantified by densitometric analysis, normalised to Ponceau red staining and expressed relative to transporter expression found in untreated cells, arbitrarily set at the value of 100% and indicated by a dotted line; they are the means ± SEM of values from four independent assays. *, p<0.05 when compared to untreated cells (ANOVA followed by a Dunnett’s post-hoc test). (B) Two independent populations of primary human hepatocytes were either untreated (control) or exposed to 25 μg/mL DEPe for 48 h. BCRP protein content was then determined by Western-blot analysis.

Western-blot analysis indicated that BCRP protein expression was significantly induced by a 2.44-fold factor by DEPe treatment in HepaRG cells (Fig. 6A). DEPe also induced BCRP expression in two independent populations of primary human hepatocytes (Fig. 6B). DEPe slightly increased protein expression of MRP2 and MRP3 by 1.45- and 1.47-fold factors, respectively, in HepaRG cells, but such inductions failed to reach a significant level (Fig. 6A). By contrast, DEPe failed to obviously alter expression of OATP1B1, OATP2B1 and P-glycoprotein/MDR1 (Fig. 6A).

**Fig 7:**
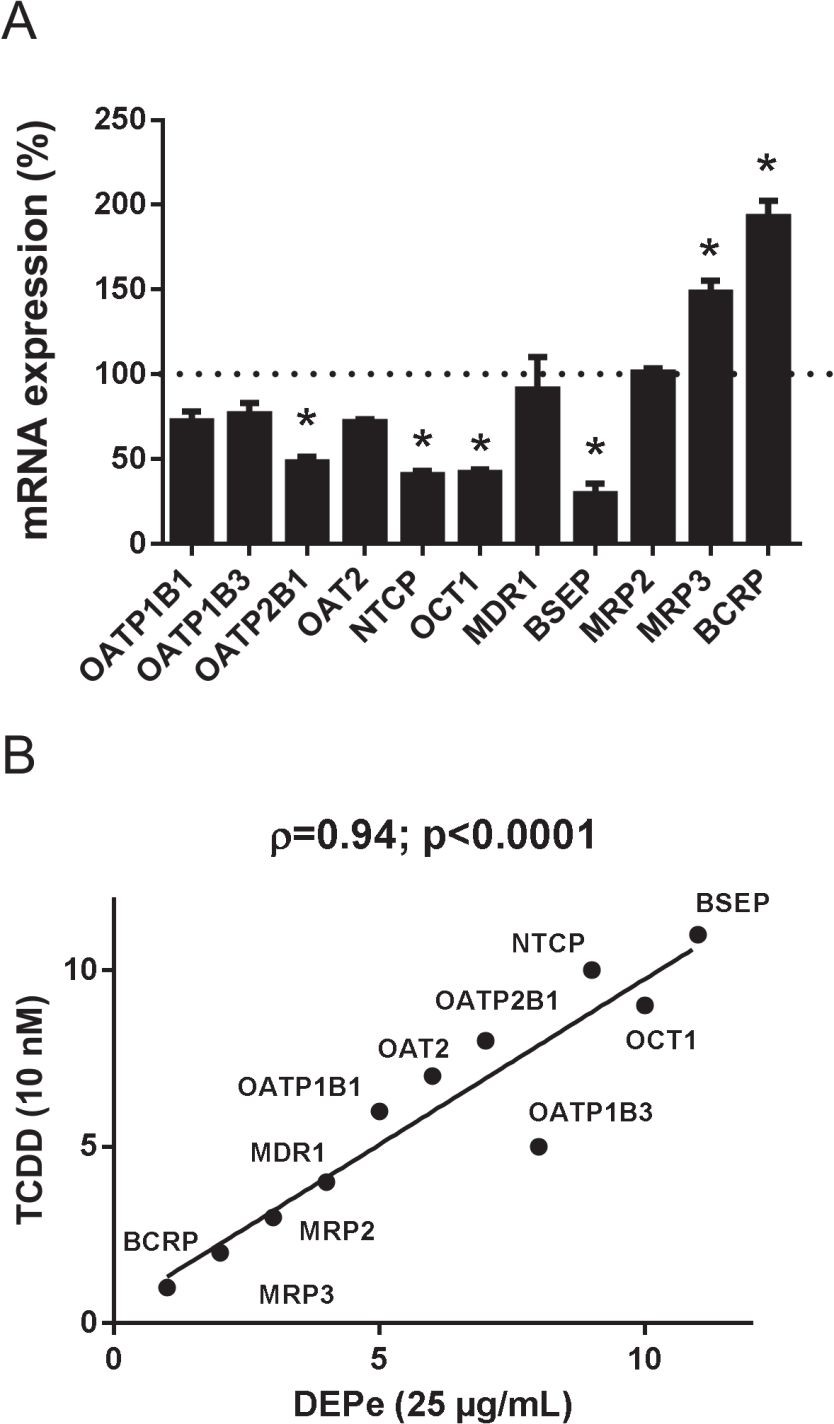
Correlation of DEPe- and TCDD-induced changes of drug transporter mRNA expression. (A) HepaRG cells were either untreated or exposed to 10 nM TCDD for 48 h. Drug transporter mRNA expression was evaluated by RT-qPCR. Data are expressed as % of transporter expression found in control untreated cells, arbitrarily set at the value of 100% and indicated by a dotted line; they are the means ± SEM of three independent assays. *, p<0.05 when compared to control cells (ANOVA followed by a Dunnett’s post-hoc test). (B) Drug transporters were ranked according to regulation of their mRNA expression in response to a 48-h treatment by 25 μg/mL DEPe or 10 nM TCDD. For this purpose, transporters were ranked for each treatment from the most induced transporter to the most repressed, from data from Fig. 5B and Fig. 7A. Correlation was analysed using the Spearman's rank correlation method. Spearman's rank coefficients (ρ) and p values are provided on the top of the correlation graph.

The fact that DEPe induced mRNA expression of CYP1A1, a reference target of the AhR pathway, indicated that this signaling way was activated by DEPe in HepaRG cells, as expected owing to the well-established presence of AhR agonists such as PAHs in DEPe [19]. This conclusion is supported by the fact that exposure to DEPe resulted in up-regulation of CYP1B1 and ALDH3A1 (S1 Fig.), two other well-known reference markers regulated by the AhR pathway [51]. Because AhR has been involved in regulation of some transporters [24, 66], its implication in DEPe-mediated regulation of drug transporters has to be considered. In this context, the exact impact of AhR activation on drug transporter expression in HepaRG cells was characterized using TCDD, a potent reference agonist of AhR, whose effects are presumed to be strictly dependent on AhR [67]. Treatment by 10 nM TCDD of HepaRG cells for 48 h induced CYP1A1, CYP1B1 and ALDH3A1 mRNA expression (S2 Fig.), indicating that the AhR signaling pathway was activated by TCDD in HepaRG cells. With respect to transporter mRNA levels, TCDD induced those of BCRP and MRP3 and repressed those of OATP2B1, NTCP, OCT1 and BSEP in HepaRG cells (Fig. 7A). This transporter expression profile caused by TCDD-mediated AhR activation was next compared to that resulting from DEPe exposure. For such a purpose, transporters were ranked from the most induced to the most repressed according to their mRNA expression level and potential correlation between TCDD and DEPe treatment with respect to drug transporter regulation was analysed using the Spearman's rank correlation method. As shown in Fig. 7B, the global effect of DEPe exposure on drug transporter expression in HepaRG cells was found to be highly correlated to that of TCDD.

## Discussion

Previous studies have shown that environmental contaminants such as DEPs can regulate detoxifying enzymes in hepatic and extra-hepatic tissues [15, 16, 68]. The data reported in the present work demonstrated that DEPe can also alter both activity and expression of human hepatic drug transporters, thus confirming that detoxifying proteins constitute molecular targets for DEPs.

DEPe was thus shown to strongly inhibit activity of the sinusoidal SLC transporters OATP1B1, OATP1B3 and OATP2B1 and of the canalicular efflux pump MRP2. NTCP and BCRP activities were also inhibited, but in a more moderate manner. These inhibitory effects of DEPe towards these transporters were rather specific because the activities of other drug transporters such as OCT1, MDR1/P-glycoprotein and BSEP, were not impacted. The mechanism by which DEPe can inhibit OATP and MRP2 activity, remains to be determined; competitive or non-competitive interactions with transporters [69] have likely to be considered. The nature of the chemical(s) present in DEPe and responsible for the inhibition of transporter activity remains also to be characterized. Involvement of PAHs found in relative notable concentrations in DEPe, such as phenanthrene, fluoranthene, benzo[b]fluoranthene, chrysene and 1-nitropyrene, can most likely be discarded. Indeed, these compounds used at a 10 μM concentration failed to alter OATP- or MRP2-mediated transport. Because inhibitions of drug transporter activity by chemicals occur through monotonic dose-dependent responses [70–72], lower concentrations of PAHs (in the 0.01–1 nM range) found in DEPe solutions inhibiting 50% of OATP1B1 or MRP2 activity (Table 1) are most likely also inactive on OAPTP1B1 and MRP2 activity. Similarly, involvement of PAH derivatives such as quinones is unlikely, owing to the lack of effects of the 1,2-naphtoquinone towards OATP and MRP2 activity. Substrates for OATPs and MRP2 are primarily organic anions and DEPe-contained chemical(s) blocking them may therefore be hypothesized to also display anionic structural features at physiological pH. The fact that the presence of an anionic group has been recently recognized as an important chemical property for inhibition of OATP1B1 or OATP1B3 in quantitative structure activity relationship (QSAR) modeling [70] supports this hypothesis. Various other physico-chemical properties required for the inhibition of OATPs or MRP2, including the value of LogD, a high number of hydrogen bond acceptors, a large molecular volume and aromaticity [70, 71], have also to be taken into account for the chemical structure of DEPe-contained chemicals inhibiting OATPs- and/or MRP2-mediated transport. In addition, it should be kept in mind that DEPe contains probably hundreds of organic chemicals and additive or synergic effects of some of these chemicals may be involved in inhibition of transporter activity by DEPe. Moreover, the role of putative metabolites formed from DEPe chemicals may have also to be considered, even if some of the cell models used in the study, including OATP-transfected CHO cells and hepatoma HuH-7 cells, are likely to poorly express xenobiotic metabolizing enzymes [34, 73].

In addition to directly inhibiting drug transporter activity, DEPe also modulates drug transporter expression. The canalicular ABC efflux BCRP was thus induced by DEPe both at mRNA and protein level in HepaRG cells. BCRP protein expression was also increased in primary human hepatocytes exposed to DEPe. The sinusoidal ABC efflux pump MRP3 was similarly up-regulated at mRNA level in HepaRG cells, but this up-regulation failed to reach significance at protein level. Such a discrepancy may be due to the relative weak mRNA induction of this transporter by DEPe or, alternatively, to a divergent regulation between transcriptional and post-transcriptional level, as already described for cytokine-mediated regulation of MRP3 [74]. In the same way, OATP2B1 was differently regulated by DEPe at mRNA (down-regulation) and protein (no change) level. Other transporters whose mRNA expression was repressed by DEPe exposure include various SLC sinusoidal transporters such as OATP1B3, OAT2, NTCP and OCT1, and the canalicular bile salt efflux pump BSEP. By contrast, expression of the sinusoidal transporter OATP1B1 and of the canalicular transporters MDR1/P-glycoprotein and MRP2 remained significantly unchanged, both at mRNA and protein levels. Overall, these DEPe-induced changes in hepatic transporter expression correspond to a repression of most of sinusoidal SLC uptake transporters and a preservation or an induction of ABC efflux pumps. They may consequently be interpreted as a protective mechanism of hepatic cells exposed to DEPe-containing chemicals, leading to decreased intracellular accumulation of potential toxic chemicals through reduction of their uptake and enhancement of their efflux. BCRP induction by DEPe likely illustrates this conclusion because BCRP up-regulation may be hypothesized to result in enhanced efflux of toxic PAH metabolite substrates for this efflux pump [24].

AhR represents a well-recognized xenobiotic-sensing receptor activated by DEPs [8, 19]. It was effectively stimulated in DEPe-exposed HepaRG cells, as demonstrated by up-regulation of the reference AhR target genes CYP1A1, CYP1B1 and ALDH3A1. The AhR signaling pathway is most likely implicated in DEPe-mediated regulations of drug transporters. Indeed, the profile of transporter expression changes in DEPe-exposed HepaRG cells is highly correlated to that resulting from treatment by the reference AhR agonist TCDD. Moreover, BCRP, which is the most up-regulated transporter by DEPe, is known to be regulated by the AhR cascade [24, 75]. In this context, various PAHs found in DEPe and known to be potent ligands for AhR, such as chrysene and benzo[b]fluoranthene [76], may be hypothesized to be the main DEPe-contained chemicals involved in transporter regulation.

The relevance of our *in vitro* findings to *in vivo* exposure to atmospheric DEPs constitutes probably a key-point that has to be clarified. In this context, it is noteworthy that the concentrations of DEPe acting on OATP or MRP2 activity or on drug transporter expression levels, that range from 1 μg/mL to 25 μg/mL, correspond to approximately 0.2 to 5 μg/cm^2^ equivalent DEP dose according to previous conversions of *in vitro* DEPe/DEP dose to DEP dose/unit surface area [56]. Importantly, such exposure levels are thought to be achieved in respiratory tracts for subjects exposed to DEPs-containing atmospheres [56], thus underlining the fact that our *in vitro* exposure conditions may be close to environmental exposure situations. Whether such relevant pulmonary exposures to DEPs may result in hepatic concentrations of DEPe-contained chemicals sufficient for acting on transporter expression or activity remains however to characterize, but may be possible. Indeed, the passage across the pulmonary alveolar-capillary barrier of ultrafine DEPs [10] and/or of some organic or inorganic DEP components as PAHs [11] has already been demonstrated, thus bringing the proof of the concept that pulmonary exposure to DEPs may result in internal exposure to DEPs-contained chemicals. This conclusion is moreover supported by the development of various systemic effects after atmospheric exposure to DEPs, including hepatic effects [12, 13].

Another key point that remains to be characterized corresponds to the potential consequences of DEP effects on hepatic transporters. Inhibition of OATPs and MRP2 by DEPs may impair hepatobiliary elimination of drug substrates for these transporters. The relevance of such putative transporter-based interactions between pollutants like DEPs and drugs is however yet unknown [77], but may have to be considered, especially for OATP1B1 and OATP1B3. Indeed, OATP1B1 and OATP1B3 handle many of the marketed drugs and are involved in many clinically important drug-drug interactions [78]. Besides drugs, endogenous substrates may also be impacted by the interaction of DEP-contained chemicals with transporters, which may contribute to DEP toxicity. For example, MRP2 has been shown to efflux glutathione conjugates out of cells and by this way to protect them against oxidative stress [79]. Inhibition of MRP2 by DEPe may therefore aggravate the well-established deleterious pro-oxidant effects of DEPs [80]. In the same way, inhibition of OATPs and MRP2 by DEPe may alter *in vivo* transport of steroid hormone substrates for these transporters, especially estrogens, which may participate to endocrine disruption caused by DEPs [81]. OATPs and MRP2 are present not only in the liver, but also in other tissues. For example, OATP2B1 is expressed at high levels in the lung [82], the primary site affected by DEPs. It is also present, as well as MRP2, at the luminal pole of endothelial cells at the blood brain-barrier [83, 84], whose integrity is compromised in response to pulmonary exposure to DEPs [85]. It may be therefore hypothesized that inhibition of OATP2B1 and MRP2 activity by DEPs may contribute to DEPe toxicity at these anatomical sites.

In summary, DEPe, used at concentrations relevant to environmental exposure situations, was shown to act as a bifunctional modulator of drug transporters, *i*.*e*., it inhibits *in vitro* activity of hepatic drug transporters such as OATPs and MRP2 and alters expression of some of them, notably BCRP. Such changes may contribute to systemic toxic effects of DEPs through impairing hepatic transport of endogenous compounds or drug substrates of these transporters.

## Supporting Information

S1 Fig Induction of CYP1B1 and ALDH3A1 mRNA expression by DEPe. Human highly-differentiated HepaRG cells were either untreated (control) or exposed to 25 μg/mL DEPe for 48 h. CYP1B1 and ALDH3A1 mRNA expressions were analysed by RT-qPCR. Data are expressed in arbitrary units relative to 18S RNA content and are the means ± SEM of three independent assays. *, p<0.05 when compared to control cells (Student’s t-test).
(TIF)

S2 Fig Induction of CYP1A1, CYP1B1 and ALDH3A1 mRNA expression by TCDD. HepaRG cells were either untreated (control) or exposed to 10 nM TCDD for 48 h. CYP1A1, CYP1B1 and ALDH3A1 mRNA expressions were evaluated by RT-qPCR. Data are expressed in arbitrary units relative to 18S RNA content and are the means ± SEM of three independent assays. *, p<0.05 when compared to control cells (Student’s t-test).
(TIF)
