# Supplementary figures and images for: Regulation of Human Hepatic Drug Transporter Activity and Expression by Diesel Exhaust Particle Extract
Source: PLoS One. 2015 Mar 24;10(3):e0121232. doi: 10.1371/journal.pone.0121232 (PMC4372591; doi:10.1371/journal.pone.0121232)

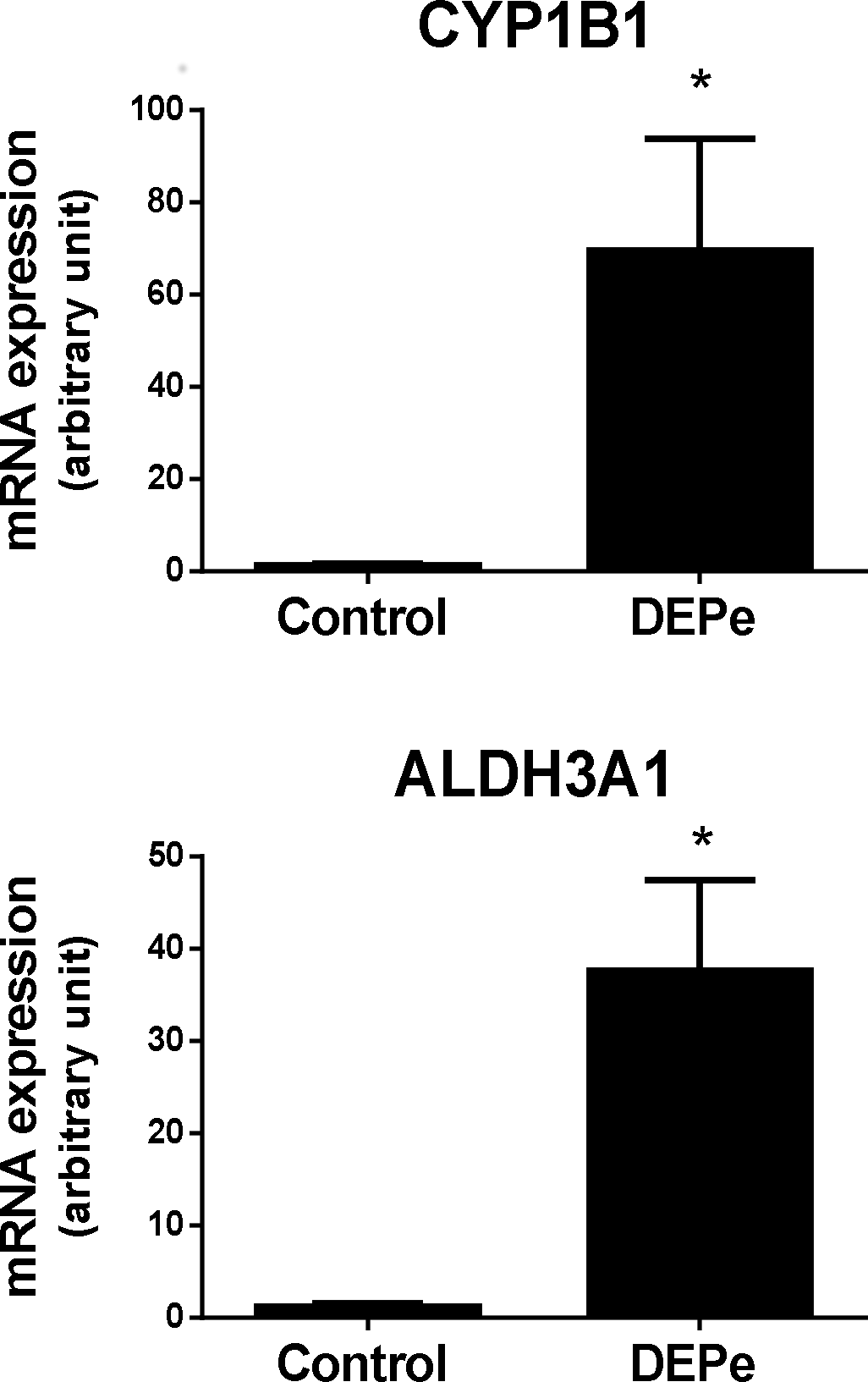

Supplement: S1 Fig — Human highly-differentiated HepaRG cells were either untreated (control) or exposed to 25 μg/mL DEPe for 48 h. CYP1B1 and ALDH3A1 mRNA expressions were analysed by RT-qPCR. Data are expressed in arbitrary units relative to 18S RNA content and are the means ± SEM of three independent assays. *, p<0.05 when compared to control cells (Student’s t-test). (TIF) [file pone.0121232.s001.tif]

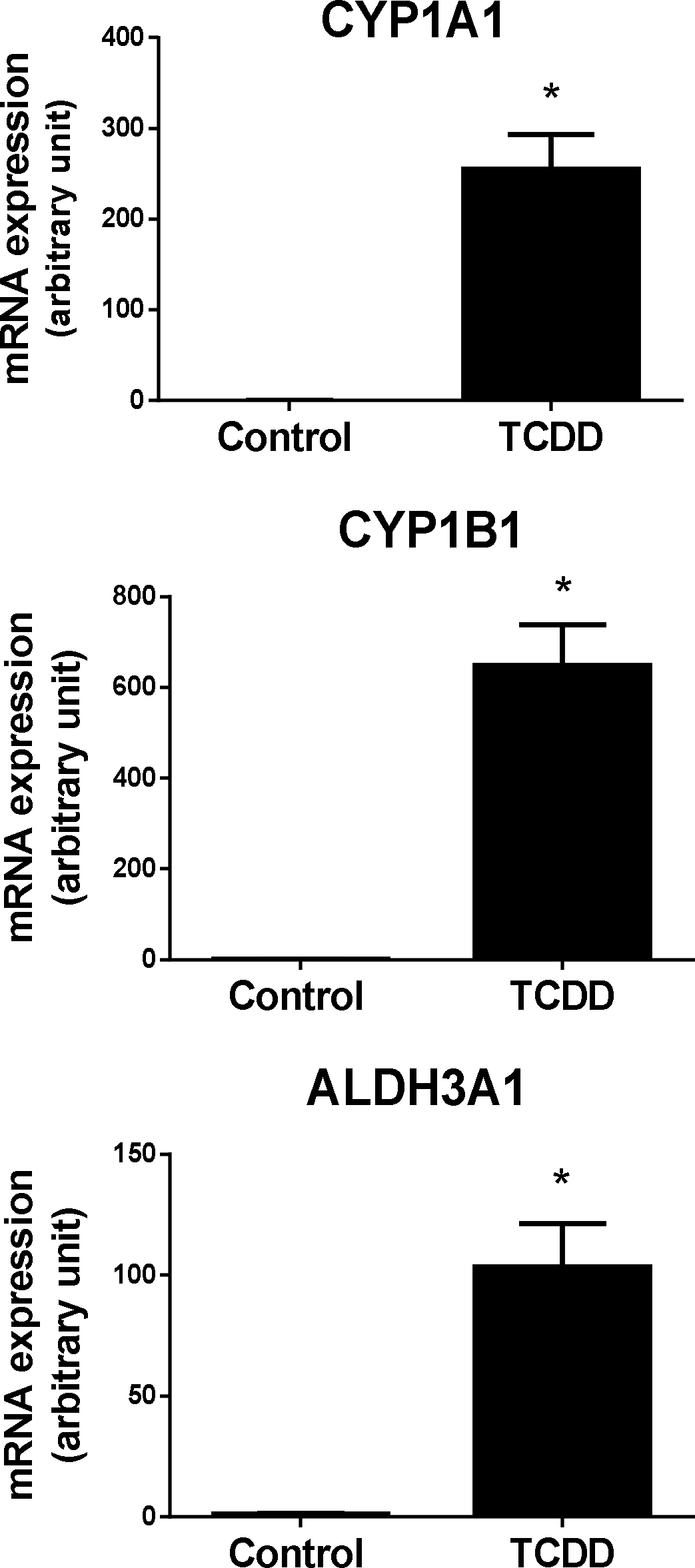

Supplement: S2 Fig — HepaRG cells were either untreated (control) or exposed to 10 nM TCDD for 48 h. CYP1A1, CYP1B1 and ALDH3A1 mRNA expressions were evaluated by RT-qPCR. Data are expressed in arbitrary units relative to 18S RNA content and are the means ± SEM of three independent assays. *, p<0.05 when compared to control cells (Student’s t-test). (TIF) [file pone.0121232.s002.tif]
